# Associations of recorded in-hospital linezolid and fluoroquinolone exposure with mortality and documented drug-induced liver injury in non-HIV tuberculous meningitis: a propensity score-weighted real-world cohort study

**DOI:** 10.3389/fmed.2026.1907126

**Published:** 2026-09-23

**Authors:** Wengao Zeng, Xiangzhi Xiao, Qing Liu, Sufen Chen, Jue Hu, Qiong Wu

**Affiliations:** 1 Department of Neurology, The Second Hospital of Changsha (West Branch of Changsha Hospital for Maternal & Child Health Care), Changsha, Hunan, China; 2 Department of Neurology, The Affiliated Changsha Central Hospital, Hengyang Medical School, University of South China, Changsha, Hunan, China; 3 Department of Neurosurgery, The Affiliated Changsha Central Hospital, Hengyang Medical School, University of South China, Changsha, Hunan, China

**Keywords:** drug-Induced liver injury, fluoroquinolone, inverse probability of treatment weighting, linezolid, real-world evidence, tuberculous meningitis

## Abstract

**Background:**

Tuberculous meningitis (TBM) remains the most severe form of tuberculosis. Linezolid- and fluoroquinolone-containing regimens are used in clinical practice, but their associations with mortality and specific toxicities in non-HIV TBM remain uncertain.

**Methods:**

We conducted a single-center retrospective cohort study of non-HIV patients with TBM hospitalized between October 2013 and January 2024. The exposures were recorded linezolid use, fluoroquinolone-class use, and combined linezolid-fluoroquinolone use during the index hospitalization. Each indexed exposure was compared with no recorded use of that exposure; for the combined analysis, the comparator was not-both exposure (either agent alone or neither agent). Outcomes were in-hospital all-cause death and drug-induced liver injury (DILI) documented during the index hospitalization. Propensity score-based inverse probability of treatment weighting and doubly robust weighted logistic regression with model-robust sandwich standard errors were used. Propensity score matching, complete-case analysis, 20-dataset multiple imputation, a sensitivity analysis restricted to age, sex, diabetes mellitus, and immunodeficiency, and subgroup interaction analyses were performed.

**Results:**

Among 1,574 patients, 187 died in hospital and 116 had DILI documented during the index hospitalization. Linezolid was recorded in 996 patients, fluoroquinolone-class therapy in 859, and both in 556. After weighting, all modeled covariates had absolute standardized mean differences below 0.10. None of the three recorded exposures was significantly associated with in-hospital death or DILI documented during the index hospitalization. Complete-case, multiple-imputation, and temporally restricted sensitivity estimates were directionally consistent with the primary results. None of the prespecified exposure-by-subgroup interactions was statistically significant after outcome-specific multiplicity adjustment.

**Conclusions:**

Recorded in-hospital linezolid, fluoroquinolone, and combined exposure were not significantly associated with in-hospital death or DILI documented during the index hospitalization in this cohort. The estimates apply to recorded inpatient exposure and DILI and do not establish the effects of treatment initiation at a defined time or the overall safety of these regimens. For DILI, the data do not establish that the liver event occurred after linezolid or fluoroquinolone initiation.

## Introduction

Tuberculous meningitis (TBM) is the most devastating manifestation of tuberculosis and remains associated with substantial mortality and neurological disability despite anti-tuberculosis therapy and adjunctive corticosteroids (1–3). Because central nervous system drug exposure is a major therapeutic concern in TBM, intensified regimens have been used in clinical practice to improve antimicrobial activity, particularly in patients with severe disease, suspected drug resistance, treatment intolerance, or inadequate response to standard therapy.

Linezolid has favorable cerebrospinal fluid penetration and activity against Mycobacterium tuberculosis, while fluoroquinolones, including levofloxacin and moxifloxacin, have been evaluated as intensified or alternative agents because of their antituberculosis activity and central nervous system exposure (4–7). However, current guidance does not establish routine linezolid- or fluoroquinolone-containing intensification as a standard strategy for all patients with drug-susceptible TBM (1, 7). Existing randomized and systematic-review evidence has not consistently demonstrated a population-level mortality benefit from intensified TBM treatment, and the role of linezolid remains less clearly defined outside selected clinical contexts (7–9). In routine practice, intensified regimens are often preferentially used in patients with greater disease severity, suspected or confirmed drug resistance, treatment intolerance, or inadequate response to standard therapy (10, 11). This treatment-selection pattern creates substantial confounding by indication in retrospective real-world analyses (12).

At the same time, intensified anti-tuberculosis regimens may increase treatment complexity and toxicity. Anti-tuberculosis drug-induced liver injury is clinically important because it may lead to treatment interruption or regimen modification (13). Contemporary cohorts document clinically important anti-tuberculosis DILI events and risk factors (14, 15). However, DILI represents only one hepatic outcome and cannot serve as a comprehensive measure of treatment safety.

In this retrospective real-world cohort of non-HIV TBM, we aimed to estimate the associations of recorded in-hospital linezolid, fluoroquinolone-class, and combined linezolid–fluoroquinolone exposure with in-hospital mortality and DILI documented during the same hospitalization. We used propensity score weighting, doubly robust outcome modeling, propensity score matching, missing-data sensitivity analyses, and prespecified exploratory subgroup analyses to address measured confounding and assess robustness.

## Materials and methods

### Study design and population

This single-center retrospective observational cohort study included patients diagnosed with TBM at Changsha Central Hospital between October 1, 2013, and January 1, 2024. Eligible patients were identified through the hospital electronic medical record system. TBM was diagnosed according to consensus clinical research criteria integrating clinical presentation, cerebrospinal fluid characteristics, neuroimaging findings, and microbiological evidence (16). Patients with documented HIV co-infection, concomitant non-tuberculous intracranial infections, or insufficient clinical data for the predefined analysis were excluded. The study was designed and reported in accordance with the STROBE statement (17). The index date was the date of hospitalization for the index TBM admission, and follow-up was restricted to the inpatient period.

The study protocol was approved by the Ethics Committee of Changsha Central Hospital (approval number: 20250122). The requirement for informed consent was waived because the study was retrospective and used anonymized data.

### Exposures

The main treatment exposures were linezolid use, fluoroquinolone-class use, and combined linezolid–fluoroquinolone use during the index TBM hospitalization. Exposure status was ascertained from inpatient medication records. The fluoroquinolone-class variable captured recorded levofloxacin and/or moxifloxacin use. Aspirin use was evaluated as an exploratory adjunctive exposure, and corticosteroid use was included as a covariate. For the binary analyses, the linezolid-unexposed group comprised fluoroquinolone-only and neither-exposed patients; the fluoroquinolone-unexposed group comprised linezolid-only and neither-exposed patients; and the combined-exposure-unexposed group comprised linezolid-only, fluoroquinolone-only, and neither-exposed patients. The exploratory aspirin analysis compared recorded aspirin use with no recorded aspirin use.

### Outcomes

The effectiveness outcome was in-hospital all-cause mortality, defined as death from any cause during the index TBM hospitalization. The prespecified safety outcome was DILI documented during hospitalization. In the source records, DILI was adjudicated from liver biochemistry using criteria aligned with consensus DILI definitions and anti-tuberculosis hepatotoxicity guidance: alanine aminotransferase (ALT) at least 5 times the upper limit of normal (ULN); alkaline phosphatase at least 2 times the ULN with hepatic origin considered; ALT at least 3 times the ULN with compatible hepatitis symptoms; or ALT at least 3 times the ULN with total bilirubin greater than 2 times the ULN. Plausible alternative causes were excluded where clinically indicated (13, 18). As a study-specific terminal-event safeguard, liver-biochemistry results obtained only during the final 24 h before death were not used to classify DILI. This rule reduced terminal-event misclassification but did not establish DILI onset relative to linezolid or fluoroquinolone. The harmonized analytic dataset retained a binary DILI indicator but not DILI event dates, complete serial biochemistry, individual-drug causality attribution, or linezolid and fluoroquinolone initiation dates. Therefore, the analysis could not establish whether documented DILI occurred after either specific exposure.

### Covariates

The prespecified covariates were age, sex, C-reactive protein, D-dimer, neutrophil count, albumin, diabetes mellitus, immunodeficiency, cerebral infarction, hydrocephalus, epilepsy, hyponatremia, drug-resistant tuberculosis, mycobacterial drug-resistance gene positivity, renal failure, pulmonary tuberculosis type, MRI classification, cerebrospinal fluid glucose, cerebrospinal fluid protein, cerebrospinal fluid white blood cell count, and corticosteroid use. For laboratory and cerebrospinal-fluid variables, the source database directly captured the first post-admission result, generally obtained within 24 h of admission. These variables were therefore early-admission measurements, but they could not be verified as pre-exposure because study-drug initiation times were unavailable. Age and sex were fixed characteristics. Other diagnoses, neurological and imaging findings, resistance indicators, and corticosteroid use were recorded during the index hospitalization; their timing relative to linezolid or fluoroquinolone initiation could not be uniformly verified. The full propensity-score covariate set is therefore described as recorded inpatient characteristics rather than uniformly pre-exposure baseline covariates.

### Statistical analysis

Patient characteristics were summarized by treatment pattern. Continuous variables were reported as medians with interquartile ranges, and categorical variables as counts with percentages. Each descriptive summary used the available observations for that variable. For the primary propensity-score and doubly robust models, missing continuous covariates were imputed with their medians and missing binary or categorical covariates with their modes. Complete-case analysis and multiple imputation by chained equations were used as sensitivity analyses. Multiple imputation used 20 imputed datasets, 20 iterations, and random seed 20260806; predictive mean matching was used for continuous covariates and logistic regression for cerebral infarction. Exposure, outcome, and all modeled covariates were included in the imputation models. Propensity scores and weights were re-estimated within each imputed dataset, and coefficients and model-robust variances were pooled using Rubin's rules (19). Trace plots and lag autocorrelation were examined as chain diagnostics. All weighted outcome models used survey-design methods with model-robust sandwich standard errors (20). The primary analysis used stabilized IPTW truncated at the 1st and 99th percentiles. Separate propensity-score models were fit for linezolid, fluoroquinolone, combined linezolid-fluoroquinolone, and aspirin. Balance was evaluated using absolute standardized mean differences (SMDs), with values below 0.10 considered acceptable (21).

Main estimates were obtained from doubly robust weighted logistic regression models including the exposure and prespecified clinical covariates. Survey-weighted quasibinomial models accommodated the weighted binary outcomes, and 95% confidence intervals and *P* values were calculated using model-robust sandwich standard errors (20). Odds ratios (ORs), 95% confidence intervals (CIs), adjusted absolute risks, and adjusted risk differences were calculated. To directly examine possible adjustment for post-exposure variables, a temporal-robustness sensitivity analysis was restricted to fixed or clearly pre-existing characteristics: age, sex, diabetes mellitus, and immunodeficiency. Because this restricted model omitted important severity-related confounders, it was interpreted only as a check on covariate-timing sensitivity and not as a less biased causal estimate. Propensity score matching (PSM) used 1:1 nearest-neighbor matching without replacement on the logit of the propensity score with a caliper of 0.2 standard deviations; matched-sample balance was assessed using standardized differences (22, 23). Matched-pair event rates, risk differences, McNemar tests, and matched-pair ORs with continuity correction were calculated. Effect modification was assessed in prespecified exploratory subgroup analyses by including exposure-by-subgroup product terms. The six prespecified subgroup variables were drug-resistant tuberculosis, mycobacterial drug-resistance gene positivity, cerebral infarction, hydrocephalus, MRI complication phenotype, and age ≥60 years. False-discovery-rate and Bonferroni adjustments were performed separately within each outcome across 18 interaction tests (three exposures by six subgroup variables). Statistical analyses were performed in R version 4.5.3. Two-sided *P* values <0.05 were considered statistically significant.

## Results

### Study population and treatment patterns

Of 1,737 patients diagnosed with TBM during the study period, 1,574 non-HIV patients were included in the final analysis after exclusions (Figure 1). The cohort included 211 children and 1,363 adults. Overall, 187 patients (11.9%) died during hospitalization and 116 patients (7.4%) had DILI documented during hospitalization. Linezolid was used in 996 patients (63.3%), fluoroquinolone-class agents in 859 (54.6%), and both linezolid and fluoroquinolone in 556 (35.3%). Corticosteroids were used in 1,535 patients (97.5%), and aspirin was used in 844 (53.6%).

**Figure 1:**
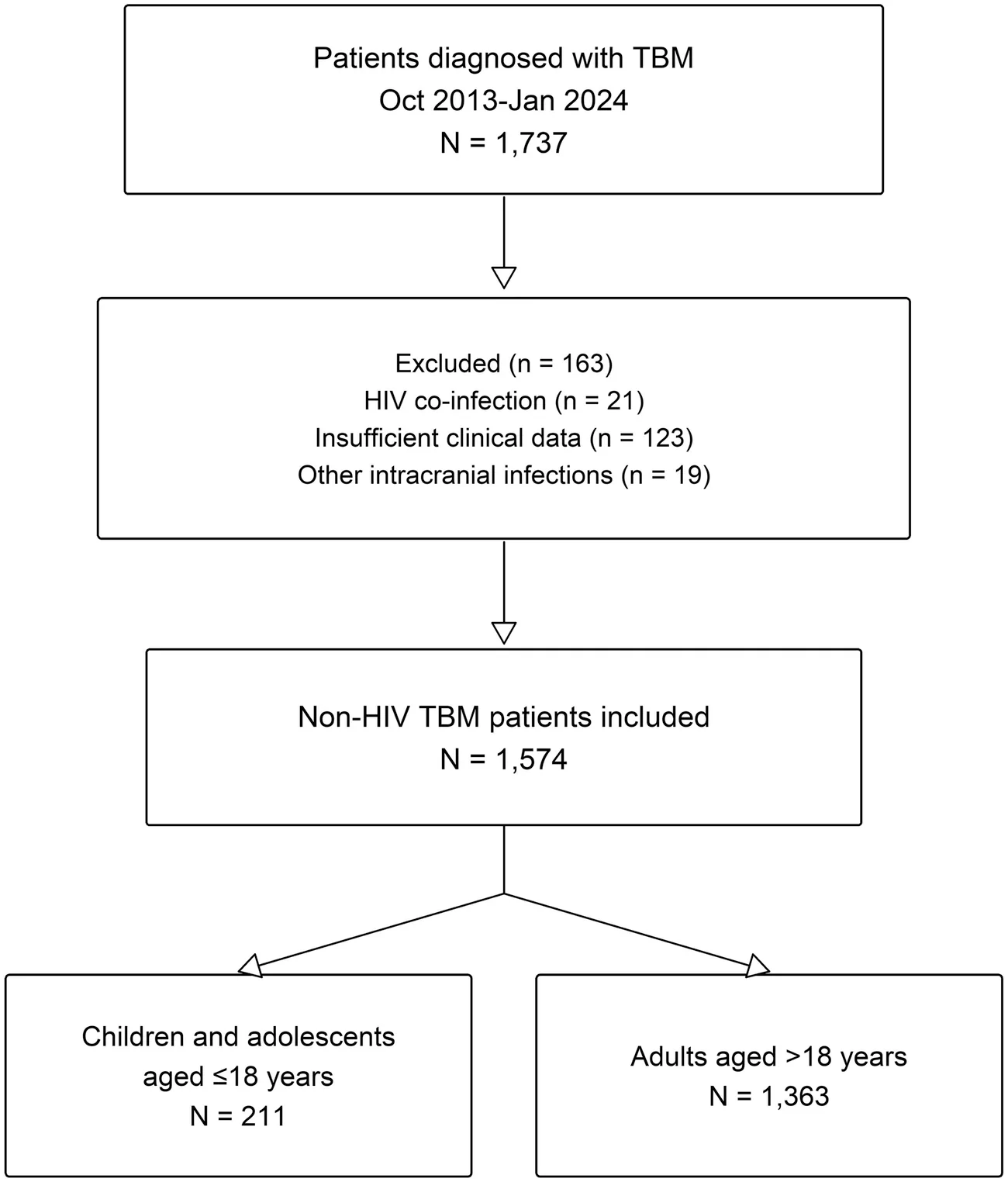
Flow diagram of patient selection for the non-HIV tuberculous meningitis cohort study. The age categories are children and adolescents aged ≤18 years and adults aged >18 years. TBM, tuberculous meningitis.

Patients were grouped into four recorded treatment patterns: both linezolid and fluoroquinolone (*n* = 556), linezolid only (*n* = 440), fluoroquinolone only (*n* = 303), and neither drug (*n* = 275). Missingness among modeled covariates was low: C-reactive protein was missing in 3 patients (0.2%), D-dimer in 11 (0.7%), neutrophil count in 19 (1.2%), albumin in 1 (0.1%), and cerebral infarction status in 1 (0.1%); all other modeled covariates were complete. Recorded characteristics were broadly similar across treatment patterns, although corticosteroid use differed because the neither group had a slightly lower corticosteroid rate (Table 1).

**Table 1:** Patient characteristics by recorded treatment pattern and all propensity-score covariates.

Characteristic	Linezolid + fluoroquinolone (*n* = 556)	Linezolid only (*n* = 440)	Fluoroquinolone only (*n* = 303)	Neither (*n* = 275)	*P* value	Missing, *n* (%)
Age, years	42.0 (26.0–58.0)	44.0 (26.0–59.0)	40.0 (24.0–59.0)	44.0 (25.0–62.5)	0.348	0 (0.0)
C-reactive protein, mg/L	5.0 (5.0–14.6)	5.0 (5.0–14.7)	5.0 (5.0–13.6)	5.0 (5.0–11.0)	0.138	3 (0.2)
D-dimer, mg/L	0.6 (0.6–0.6)	0.6 (0.6–0.6)	0.6 (0.6–0.6)	0.6 (0.6–0.6)	0.098	11 (0.7)
Neutrophil count, × 10^9^/L	3.5 (2.6–3.5)	3.5 (2.6–4.3)	3.5 (2.6–4.0)	3.5 (2.6–3.6)	0.559	19 (1.2)
Albumin, g/L	35.0 (31.7–38.3)	35.0 (32.5–38.1)	35.0 (32.4–38.5)	35.0 (32.0–39.0)	0.894	1 (0.1)
CSF glucose, mmol/L	2.2 (2.2–3.1)	2.2 (2.2–3.1)	2.2 (2.2–3.4)	2.3 (2.2–3.4)	0.245	0 (0.0)
CSF protein, g/L	0.9 (0.6–1.3)	0.9 (0.6–1.3)	0.9 (0.6–1.5)	0.9 (0.5–1.7)	0.941	0 (0.0)
CSF WBC count, × 10^6^/L	10.0 (10.0–40.0)	10.0 (10.0–52.0)	10.0 (10.0–100.0)	10.0 (6.0–80.0)	0.174	0 (0.0)
Female sex	361 (64.9)	302 (68.6)	189 (62.4)	183 (66.5)	0.333	0 (0.0)
Pulmonary tuberculosis type: Absent	133 (23.9)	97 (22.0)	69 (22.8)	48 (17.5)	0.140	0 (0.0)
Hematogenous disseminated	166 (29.9)	124 (28.2)	106 (35.0)	94 (34.2)		
Primary pulmonary tuberculosis	22 (4.0)	16 (3.6)	9 (3.0)	6 (2.2)		
Secondary pulmonary tuberculosis	231 (41.5)	203 (46.1)	119 (39.3)	126 (45.8)		
Tuberculous pleurisy	4 (0.7)	0 (0.0)	0 (0.0)	1 (0.4)		
MRI classification: Negative	80 (14.4)	69 (15.7)	35 (11.6)	42 (15.3)	0.591	0 (0.0)
Parenchymal type	390 (70.1)	289 (65.7)	216 (71.3)	173 (62.9)		
Meningeal type	47 (8.5)	50 (11.4)	29 (9.6)	34 (12.4)		
Complication type	27 (4.9)	20 (4.5)	16 (5.3)	18 (6.5)		
Spinal arachnoiditis type	12 (2.2)	12 (2.7)	7 (2.3)	8 (2.9)		
Diabetes mellitus	34 (6.1)	23 (5.2)	12 (4.0)	14 (5.1)	0.603	0 (0.0)
Immunodeficiency	157 (28.2)	127 (28.9)	78 (25.7)	90 (32.7)	0.316	0 (0.0)
Cerebral infarction	103 (18.5)	72 (16.4)	41 (13.5)	49 (17.9)	0.286	1 (0.1)
Hydrocephalus	36 (6.5)	39 (8.9)	22 (7.3)	22 (8.0)	0.548	0 (0.0)
Epilepsy	34 (6.1)	17 (3.9)	20 (6.6)	15 (5.5)	0.335	0 (0.0)
Hyponatremia	157 (28.2)	116 (26.4)	88 (29.0)	81 (29.5)	0.790	0 (0.0)
Drug-resistant tuberculosis	36 (6.5)	26 (5.9)	23 (7.6)	13 (4.7)	0.540	0 (0.0)
Mycobacterial drug-resistance gene positivity	16 (2.9)	17 (3.9)	12 (4.0)	10 (3.6)	0.797	0 (0.0)
Renal failure	143 (25.7)	119 (27.0)	82 (27.1)	68 (24.7)	0.884	0 (0.0)
Corticosteroid use	541 (97.3)	437 (99.3)	301 (99.3)	256 (93.1)	<0.001	0 (0.0)
Aspirin use	299 (53.8)	238 (54.1)	176 (58.1)	131 (47.6)	0.092	0 (0.0)

Values are median (IQR) or *n* (%). Missing counts are reported for the full cohort (*N* = 1,574). All 21 prespecified propensity-score covariates are shown; aspirin is shown descriptively as an exploratory exposure. *P* values are descriptive only and were not used for variable selection. CSF, cerebrospinal fluid; MRI, magnetic resonance imaging; TB, tuberculosis; WBC, white blood cell.

### Primary IPTW and doubly robust analyses

The propensity score models showed good overlap and stable stabilized IPTW weights. Effective sample sizes remained close to the original cohort size for all exposures. After weighting, the maximum absolute SMD was 0.021 for linezolid, 0.026 for fluoroquinolone exposure, 0.065 for combined linezolid–fluoroquinolone exposure, and 0.043 for aspirin; no covariate had a post-weighting SMD above 0.10.

In doubly robust survey-weighted logistic regression, recorded in-hospital linezolid, fluoroquinolone, and combined linezolid-fluoroquinolone exposure were not significantly associated with in-hospital mortality. The corresponding ORs were 0.87 (95% CI 0.62–1.21; *P* = 0.398), 0.96 (95% CI 0.69–1.33; *P* = 0.816), and 0.87 (95% CI 0.61–1.23; *P* = 0.426), respectively. The corresponding ORs for DILI documented during the index hospitalization were 0.88 (95% CI 0.59–1.32; *P* = 0.543), 1.14 (95% CI 0.76–1.69; *P* = 0.526), and 0.95 (95% CI 0.63–1.45; *P* = 0.826). Adjusted absolute risks and risk differences are presented in Table 2, and the ORs and 95% CIs are displayed in Figure 2. Covariate balance before and after IPTW for the three main exposures is shown in Figure 3. Aspirin, analyzed separately as an exploratory exposure, was not significantly associated with either outcome.

**Table 2:** Main IPTW and doubly robust associations with in-hospital death and DILI documented during the index hospitalization.

Exposure	Outcome	IPTW/DR OR (95% CI)	Adjusted risk, % exposed	Adjusted risk, % unexposed	Adjusted risk difference, pp	*P* value	ESS
Linezolid	In-hospital death	0.87 (0.62–1.21)	11.25	12.60	−1.34	0.398	1,548.5
Linezolid	DILI	0.88 (0.59–1.32)	7.05	7.84	−0.79	0.543	1,548.5
Fluoroquinolone	In-hospital death	0.96 (0.69–1.33)	11.71	12.08	−0.37	0.816	1,541.1
Fluoroquinolone	DILI	1.14 (0.76–1.69)	7.59	6.80	+0.79	0.526	1,541.1
Linezolid + fluoroquinolone	In-hospital death	0.87 (0.61–1.23)	11.09	12.41	−1.32	0.426	1,553.5
Linezolid + fluoroquinolone	DILI	0.95 (0.63–1.45)	7.25	7.55	−0.29	0.826	1,553.5
Aspirin (exploratory)	In-hospital death	1.21 (0.87–1.69)	13.00	11.17	+1.83	0.255	1,537.5
Aspirin (exploratory)	DILI	0.98 (0.65–1.45)	7.45	7.61	−0.16	0.902	1,537.5

ORs were estimated using stabilized IPTW followed by covariate-adjusted doubly robust survey-weighted logistic regression with model-robust sandwich standard errors. Risk differences are expressed in percentage points (exposed minus unexposed). DILI, drug-induced liver injury; DR, doubly robust; ESS, effective sample size; IPTW, inverse probability of treatment weighting; OR, odds ratio; pp, percentage points. Unexposed meant no recorded use of the indexed exposure: no linezolid (fluoroquinolone only or neither), no fluoroquinolone (linezolid only or neither), not both drugs (either agent alone or neither), or no aspirin, respectively. Thus, the combined-exposure estimate is both versus not both, rather than both versus neither. DILI denotes DILI documented during the index hospitalization; temporal sequence relative to either study drug was unavailable.

**Figure 2:**
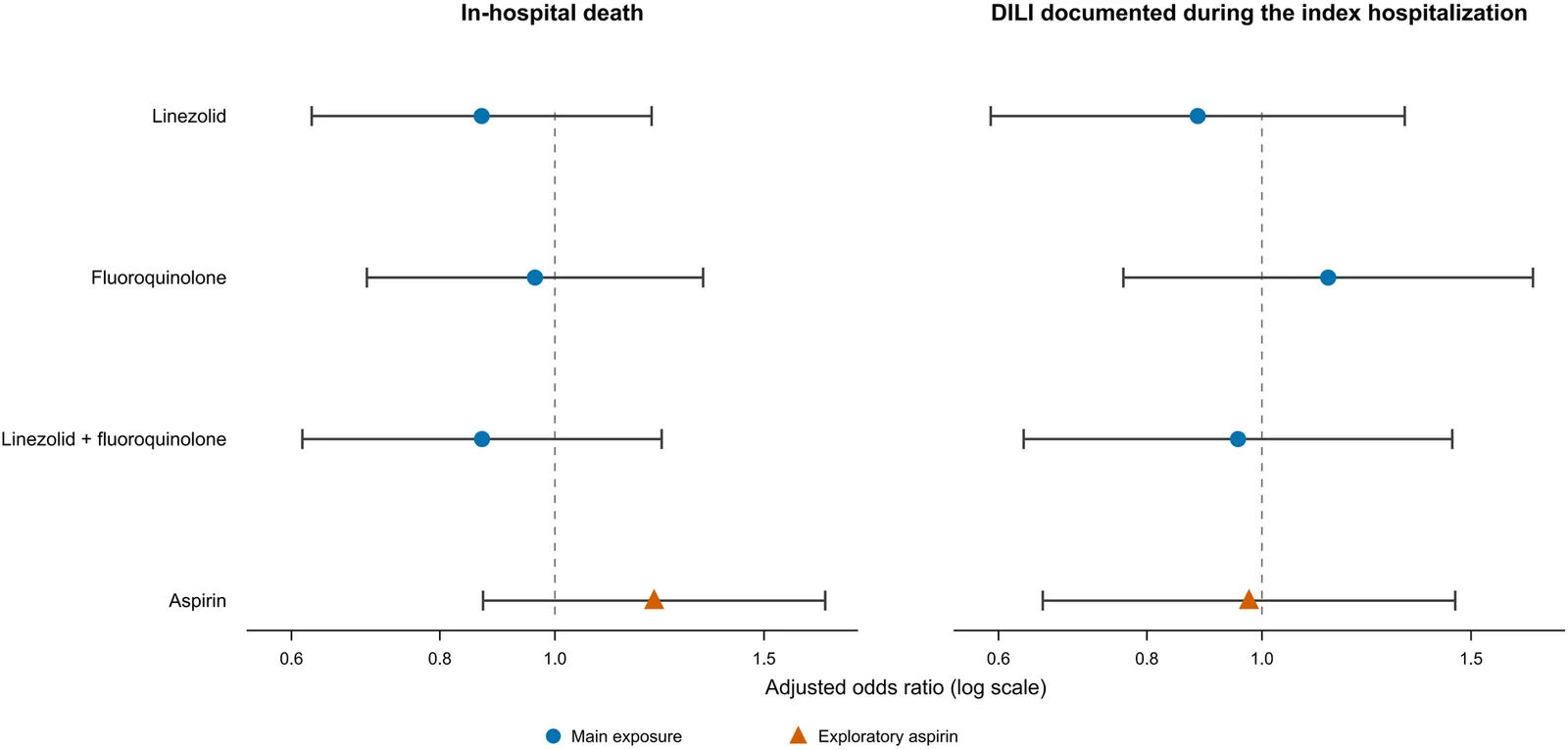
Forest plot of IPTW and doubly robust associations with in-hospital death and DILI documented during the index hospitalization. Horizontal lines indicate model-robust 95% confidence intervals and the dashed vertical line indicates OR = 1. Blue circles denote prespecified main exposures; orange triangles denote exploratory aspirin. Numeric estimates are reported in Table 2. The exposure variables do not establish that study-drug initiation preceded DILI.

**Figure 3:**
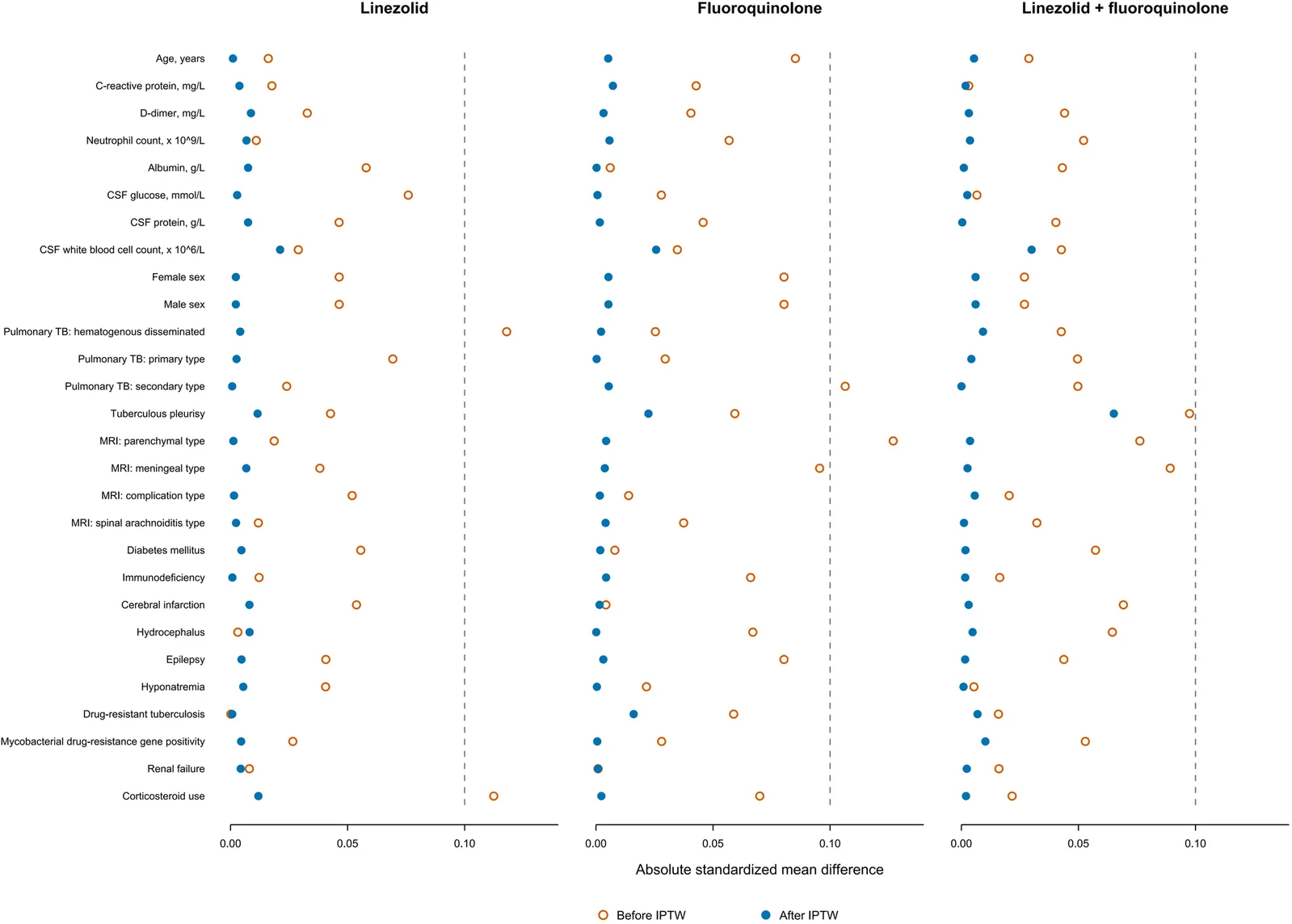
Covariate balance before and after inverse probability of treatment weighting for the three main exposures. Orange open circles show pre-weighting values and blue filled circles show post-weighting values. The same clinical covariate order and *x*-axis scale are used in all three panels. The dashed line indicates the prespecified absolute SMD threshold of 0.10.

### Observed event rates

Crude in-hospital mortality was 10.8% in the combined linezolid–fluoroquinolone group, 11.8% in the linezolid-only group, 13.5% in the fluoroquinolone-only group, and 12.4% in patients receiving neither drug. The proportions with DILI documented during hospitalization were 7.0%, 6.8%, 8.9%, and 7.3%, respectively.

### Sensitivity and subgroup analyses

PSM results were directionally consistent with the primary IPTW/DR analysis (Table 3). In the sensitivity analysis restricted to age, sex, diabetes mellitus, and immunodeficiency, the ORs for death were 0.86 (95% CI 0.63–1.18) for linezolid, 1.01 (0.74–1.38) for fluoroquinolone, and 0.87 (0.62–1.20) for combined exposure; the corresponding ORs for DILI documented during the index hospitalization were 0.85 (0.58–1.25), 1.11 (0.75–1.62), and 0.93 (0.62–1.39). None was statistically significant. Complete-case analysis included 1,555 patients and yielded death ORs of 0.83 (0.60–1.15), 0.94 (0.68–1.31), and 0.85 (0.60–1.21), and DILI ORs of 0.87 (0.58–1.30), 1.18 (0.79–1.77), and 0.98 (0.64–1.49), respectively. Multiple-imputation estimates were also similar: death ORs were 0.87 (0.63–1.21), 0.96 (0.69–1.33), and 0.87 (0.62–1.23), and DILI ORs were 0.89 (0.59–1.32), 1.14 (0.77–1.69), and 0.96 (0.63–1.45), respectively (Table 4).

**Table 3:** PSM sensitivity analyses and selected robust subgroup interaction analysis.

Analysis	Exposure or subgroup contrast	Outcome	Estimate	*P* value/adjusted *P* value	Details
PSM matched analysis	Linezolid	In-hospital death	0.85 (0.59–1.21)	0.411	572 pairs; event rates 11.2% vs. 12.9%
PSM matched analysis	Linezolid	DILI	0.83 (0.53–1.31)	0.488	572 pairs; event rates 6.8% vs. 8.0%
PSM matched analysis	Fluoroquinolone	In-hospital death	1.03 (0.75–1.41)	0.935	682 pairs; event rates 12.2% vs. 11.9%
PSM matched analysis	Fluoroquinolone	DILI	1.19 (0.81–1.76)	0.426	682 pairs; event rates 8.1% vs. 6.7%
PSM matched analysis	Linezolid + fluoroquinolone	In-hospital death	0.81 (0.56–1.17)	0.303	547 pairs; event rates 11.0% vs. 13.2%
PSM matched analysis	Linezolid + fluoroquinolone	DILI	0.90 (0.58–1.41)	0.734	547 pairs; event rates 7.1% vs. 7.9%
Selected subgroup interaction	Linezolid × drug-resistant tuberculosis	DILI	*P* for interaction = 0.031	FDR *P* = 0.555; Bonferroni *P* = 0.559	Exploratory; not significant after multiplicity adjustment

Footnote: Matched-pair ORs use 0.5 continuity correction for discordant pairs. PSM analyses are sensitivity analyses only. The subgroup interaction analysis used a survey-weighted model with a model-robust sandwich standard error; multiplicity was adjusted within the 18 DILI interaction tests. DILI, drug-induced liver injury; FDR, false discovery rate; OR, odds ratio; PSM, propensity score matching.

**Table 4:** Sensitivity analyses for covariate timing and missing data.

**Exposure**	**Outcome**	**Primary analysis, OR (95% CI)**	**Fixed or clearly pre-existing covariates, OR (95% CI)**	**Complete case, OR (95% CI)**	**Multiple imputation, OR (95% CI)**
Linezolid	In-hospital death	0.87 (0.62–1.21)	0.86 (0.63–1.18)	0.83 (0.60–1.15)	0.87 (0.63–1.21)
Linezolid	DILI	0.88 (0.59–1.32)	0.85 (0.58–1.25)	0.87 (0.58–1.30)	0.89 (0.59–1.32)
Fluoroquinolone	In-hospital death	0.96 (0.69–1.33)	1.01 (0.74–1.38)	0.94 (0.68–1.31)	0.96 (0.69–1.33)
Fluoroquinolone	DILI	1.14 (0.76–1.69)	1.11 (0.75–1.62)	1.18 (0.79–1.77)	1.14 (0.77–1.69)
Linezolid + fluoroquinolone	In-hospital death	0.87 (0.61–1.23)	0.87 (0.62–1.20)	0.85 (0.60–1.21)	0.87 (0.62–1.23)
Linezolid + fluoroquinolone	DILI	0.95 (0.63–1.45)	0.93 (0.62–1.39)	0.98 (0.64–1.49)	0.96 (0.63–1.45)

Primary analyses used median/mode imputation (*N* = 1,574). The fixed or clearly pre-existing covariate analysis included age, sex, diabetes mellitus, and immunodeficiency and is interpreted only as a temporal-robustness check. Complete-case analyses included 1,555 patients. Multiple imputation used 20 chained-equation datasets, 20 iterations, seed 20260806, and Rubin's rules. All estimates used model-robust sandwich variances. DILI, drug-induced liver injury; OR, odds ratio.

All 36 prespecified exposure-by-subgroup interaction models converged. None was statistically significant after adjustment within its outcome-specific family of 18 tests (Supplementary Table S1). For DILI documented during the index hospitalization, the linezolid-by-drug-resistant-tuberculosis interaction yielded an unadjusted *P* for interaction of 0.031, an FDR-adjusted *P* of 0.555, and a Bonferroni-adjusted *P* of 0.559 (Table 3; Supplementary Table S1). Six stratum-specific models in the drug-resistance-gene-positive subgroup did not converge and are reported as not estimable; the corresponding interaction models did converge.

## Discussion

In this retrospective cohort of non-HIV TBM, recorded in-hospital linezolid, fluoroquinolone, and combined exposure were not significantly associated with in-hospital mortality after propensity-score weighting and doubly robust adjustment. These exposures were also not significantly associated with DILI documented during the index hospitalization. Complete-case, multiple-imputation, temporally restricted, and PSM analyses were directionally consistent. The results describe associations with ever-recorded inpatient exposure and do not estimate the causal effect of starting an intensified regimen at a defined clinical time point. For DILI documented during the index hospitalization, the data establish neither that exposure preceded the liver event nor that either study drug caused the event.

The absence of a statistically significant mortality association does not establish lack of benefit. Linezolid and fluoroquinolones have pharmacokinetic and microbiological rationale in TBM (4, 5, 24), but treatment selection in routine care is influenced by severity, suspected resistance, intolerance, and response to initial therapy (1, 10–12). Propensity methods can balance recorded covariates but cannot eliminate confounding from unavailable or imprecisely measured factors, including standardized TBM grade, neurological status, clinician indication, adherence, detailed susceptibility, and treatment timing. Residual confounding by indication may therefore have obscured benefit or harm, and the confidence intervals remain compatible with clinically relevant effects in either direction.

Treatment timing is a particularly important threat to validity. Patients were classified as exposed if they received a study drug at any point during hospitalization, even when treatment may have been initiated after deterioration or inadequate response. Patients had to remain alive and hospitalized long enough to receive a later treatment, whereas rescue use may preferentially identify more severely ill patients. Without initiation dates, early-exposure or landmark analyses and time-varying treatment models were not possible. Immortal-time-related bias, reverse causation, and time-dependent confounding may consequently operate in different directions. The neutral associations should therefore not be compared directly with protocol-defined treatment effects from randomized trials, which specify assignment, dose, timing, and follow-up (8, 9).

The DILI analysis addresses a specific hepatic outcome documented during the index hospitalization, not overall safety. Source adjudication used liver-biochemistry criteria and excluded results obtained only during the final 24 h before death, but the harmonized analysis retained neither DILI onset dates nor linezolid or fluoroquinolone initiation dates. Thus, neither the source rule nor the binary analysis can establish that either study drug preceded or caused a documented DILI event. No statistically significant association was detected, and the low adjusted risk differences were consistent across sensitivity analyses. However, the dataset did not systematically capture other clinically important adverse effects, including linezolid-associated cytopenias, peripheral or optic neuropathy, and lactic acidosis, or fluoroquinolone-associated QT prolongation, dysglycemia, tendinopathy, and central nervous system effects (1, 10). The absence of an association with DILI documented during the index hospitalization must not be interpreted as proof of overall safety or as justification to reduce laboratory and clinical monitoring.

The composite fluoroquinolone exposure also limits clinical interpretation. Levofloxacin and moxifloxacin differ in pharmacokinetics, central nervous system penetration, anti-tuberculosis activity, dosing, interactions, and toxicity profiles (6, 10, 22). Dataset harmonization combined them, and reliable agent-specific dose and duration were unavailable. Consequently, the class-level estimate cannot be assigned to either agent or used to infer that their effects are interchangeable. In addition, the combined-exposure estimate compared patients receiving both drugs with a heterogeneous not-both group (either agent alone or neither); it does not estimate the incremental effect of adding one drug to the other.

IPTW diagnostics support balance for the measured covariates: stabilized weights were not extreme, effective sample sizes remained close to the cohort size, and all post-weighting SMDs were below 0.10 (21, 25). These diagnostics show balance in recorded covariates but do not establish that all covariates preceded treatment exposure. Doubly robust models combined weighting with outcome regression (26). Complete-case and multiple-imputation results were similar to the primary simple-imputation estimates, which reduces concern that the low level of covariate missingness drove the findings. Nevertheless, no propensity-based method or missing-data procedure can correct unmeasured confounding, misclassified exposure timing, or informative missingness that is not adequately represented in the imputation model (27, 28).

The subgroup analyses were exploratory. The interaction tests did not identify statistically significant effect modification for either outcome after outcome-specific multiplicity adjustment. The nominal DILI interaction for linezolid by drug-resistant tuberculosis did not remain statistically significant after either adjustment. Six stratum-specific models in the drug-resistance-gene-positive subgroup did not converge and were reported as not estimable rather than as extreme ORs. These findings do not identify a treatment-responsive subgroup (29, 30).

Strengths include the large non-HIV TBM cohort, prespecified covariates, formal balance diagnostics, doubly robust modeling with model-robust sandwich variance, adjusted absolute risks, PSM, complete-case and multiple-imputation sensitivity analyses, a temporally restricted covariate sensitivity analysis, and fully reported exploratory interaction analyses. The study treated documented DILI as an outcome and did not include it as a propensity-score covariate.

Several limitations are central to interpretation. First, this single-center retrospective study remains vulnerable to residual and unmeasured confounding and has limited generalizability. Standardized TBM severity grade, baseline Glasgow Coma Scale score, neurological disability, clinician treatment rationale, adherence, and quantitative imaging severity were not uniformly available. Second, exact treatment initiation, relation to admission and clinical deterioration, dose, duration, modification, adherence, and plasma or cerebrospinal-fluid concentrations were unavailable. Ever-exposed classification could therefore produce exposure misclassification, immortal-time-related bias, reverse causation, and time-dependent confounding; timing-sensitive exposure analyses were not feasible. The laboratory and cerebrospinal-fluid covariates were the first post-admission results, generally obtained within 24 h, but study-drug initiation times were unavailable, so even these early-admission measures could not be confirmed as pre-exposure. Some neurological complications, microbiological or resistance findings, imaging classifications, and corticosteroid use may have been identified or initiated after a study drug. Adjustment for post-exposure variables therefore cannot be excluded. Estimates from the model restricted to age, sex, diabetes mellitus, and immunodeficiency were directionally consistent with the primary estimates, but this analysis omitted important severity-related confounders and should be interpreted only as a temporal-robustness check, not as a less biased causal estimate. Third, a validated admission-date or calendar-year field was absent, so secular changes in diagnosis, supportive care, resistance, prescribing, and outcome over the 2013–2024 period could not be adjusted. Fourth, harmonized fluoroquinolone exposure did not permit reliable separation of levofloxacin from moxifloxacin. Fifth, DILI source adjudication used liver biochemistry and excluded results obtained only in the final 24 h before death, but the harmonized dataset lacked event dates, uniformly detailed serial biochemistry, and individual-drug causality; the exposure-to-DILI sequence could not be determined, and non-hepatic toxicities were not systematically captured. Sixth, the component fields underlying the historical exclusion category “insufficient clinical data” were not retained in the analytic dataset and could not be reconstructed, so selection related to that exclusion cannot be fully assessed. Finally, PSM balance was less optimal than IPTW for some exposures, and subgroup estimates were exploratory and sometimes sparse.

In summary, recorded in-hospital linezolid, fluoroquinolone, and combined exposure were not significantly associated with in-hospital mortality or DILI documented during the index hospitalization. These findings are compatible with several clinical effects and cannot establish or exclude benefit from an agent started at a specific time, nor can the DILI analysis establish that DILI documented during the index hospitalization followed a study drug or establish overall safety. Prospective or time-resolved studies are needed.

## Conclusions

Recorded in-hospital linezolid, fluoroquinolone, and combined exposure were not significantly associated with in-hospital mortality or DILI documented during the index hospitalization after propensity-score weighting and doubly robust adjustment. The estimates concern ever-recorded inpatient exposure rather than treatment initiated at a defined time and do not establish overall regimen safety. For DILI documented during the index hospitalization, the data do not establish temporal sequence relative to either study drug.

## Data Availability

The original contributions presented in the study are included in the article/Supplementary Material, further inquiries can be directed to the corresponding author.
