# Supplementary material for: Associations of recorded in-hospital linezolid and fluoroquinolone exposure with mortality and documented drug-induced liver injury in non-HIV tuberculous meningitis: a propensity score-weighted real-world cohort study
Source: Front Med (Lausanne). 2026 Sep 23;13:1907126. doi: 10.3389/fmed.2026.1907126 (PMC13642016; doi:10.3389/fmed.2026.1907126)
Supplement: Supplementary file 1 [file Table1.docx]

**Supplementary Table S1. Prespecified subgroup analyses with model-robust variance**

Associations of Recorded In-Hospital Linezolid and Fluoroquinolone Exposure With Mortality and Documented Drug-Induced Liver Injury in Non-HIV Tuberculous Meningitis: A Propensity Score-Weighted Real-World Cohort Study

Stratum-specific estimates are exploratory. Interaction P values came from survey-weighted interaction models with model-robust sandwich standard errors. FDR and Bonferroni adjustments were performed separately within each outcome across 18 interaction tests (three exposures by six subgroup variables). All 36 interaction models converged. Six stratum-specific models in the drug-resistance-gene-positive subgroup did not converge and are reported as NE.

**Supplementary Table S1a. In-hospital mortality**

| **Exposure** | **Subgroup** | **Level** | **n** | **Events** | **OR (95% CI)** | **P interaction** | **FDR P** | **Bonferroni P** |
| --- | --- | --- | --- | --- | --- | --- | --- | --- |
| Linezolid | Drug-resistant tuberculosis | Absent | 1476 | 172 | 0.94 (0.66–1.32) | 0.147 | 0.824 | 1.000 |
| Linezolid | Drug-resistant tuberculosis | Present | 98 | 15 | 0.17 (0.02–1.29) | 0.147 | 0.824 | 1.000 |
| Linezolid | Drug-resistance gene positivity | Absent | 1519 | 177 | 0.90 (0.64–1.27) | 0.332 | 0.824 | 1.000 |
| Linezolid | Drug-resistance gene positivity | Present | 55 | 10 | NE | 0.332 | 0.824 | 1.000 |
| Linezolid | Cerebral infarction | Absent | 1309 | 150 | 0.83 (0.57–1.19) | 0.412 | 0.824 | 1.000 |
| Linezolid | Cerebral infarction | Present | 265 | 37 | 1.23 (0.51–2.99) | 0.412 | 0.824 | 1.000 |
| Linezolid | Hydrocephalus | Absent | 1455 | 156 | 0.82 (0.58–1.18) | 0.374 | 0.824 | 1.000 |
| Linezolid | Hydrocephalus | Present | 119 | 31 | 1.70 (0.51–5.68) | 0.374 | 0.824 | 1.000 |
| Linezolid | MRI complication phenotype | No | 1493 | 172 | 0.85 (0.61–1.20) | 0.671 | 0.943 | 1.000 |
| Linezolid | MRI complication phenotype | Yes | 81 | 15 | 0.41 (0.04–4.59) | 0.671 | 0.943 | 1.000 |
| Linezolid | Age ≥60 years | <60 years | 1181 | 134 | 0.87 (0.59–1.30) | 0.943 | 0.943 | 1.000 |
| Linezolid | Age ≥60 years | ≥60 years | 393 | 53 | 0.86 (0.42–1.74) | 0.943 | 0.943 | 1.000 |
| Fluoroquinolone | Drug-resistant tuberculosis | Absent | 1476 | 172 | 1.01 (0.72–1.41) | 0.387 | 0.824 | 1.000 |
| Fluoroquinolone | Drug-resistant tuberculosis | Present | 98 | 15 | 0.67 (0.09–5.31) | 0.387 | 0.824 | 1.000 |
| Fluoroquinolone | Drug-resistance gene positivity | Absent | 1519 | 177 | 0.96 (0.68–1.34) | 0.854 | 0.943 | 1.000 |
| Fluoroquinolone | Drug-resistance gene positivity | Present | 55 | 10 | NE | 0.854 | 0.943 | 1.000 |
| Fluoroquinolone | Cerebral infarction | Absent | 1309 | 150 | 0.95 (0.66–1.37) | 0.760 | 0.943 | 1.000 |
| Fluoroquinolone | Cerebral infarction | Present | 265 | 37 | 0.96 (0.42–2.22) | 0.760 | 0.943 | 1.000 |
| Fluoroquinolone | Hydrocephalus | Absent | 1455 | 156 | 1.04 (0.73–1.48) | 0.374 | 0.824 | 1.000 |
| Fluoroquinolone | Hydrocephalus | Present | 119 | 31 | 0.56 (0.21–1.50) | 0.374 | 0.824 | 1.000 |
| Fluoroquinolone | MRI complication phenotype | No | 1493 | 172 | 0.99 (0.70–1.39) | 0.510 | 0.917 | 1.000 |
| Fluoroquinolone | MRI complication phenotype | Yes | 81 | 15 | 0.06 (<0.01–0.44) | 0.510 | 0.917 | 1.000 |
| Fluoroquinolone | Age ≥60 years | <60 years | 1181 | 134 | 0.81 (0.55–1.20) | 0.126 | 0.824 | 1.000 |
| Fluoroquinolone | Age ≥60 years | ≥60 years | 393 | 53 | 1.42 (0.73–2.74) | 0.126 | 0.824 | 1.000 |
| Linezolid + fluoroquinolone | Drug-resistant tuberculosis | Absent | 1476 | 172 | 0.93 (0.65–1.33) | 0.235 | 0.824 | 1.000 |
| Linezolid + fluoroquinolone | Drug-resistant tuberculosis | Present | 98 | 15 | 0.28 (0.03–2.47) | 0.235 | 0.824 | 1.000 |
| Linezolid + fluoroquinolone | Drug-resistance gene positivity | Absent | 1519 | 177 | 0.87 (0.61–1.25) | 0.827 | 0.943 | 1.000 |
| Linezolid + fluoroquinolone | Drug-resistance gene positivity | Present | 55 | 10 | NE | 0.827 | 0.943 | 1.000 |
| Linezolid + fluoroquinolone | Cerebral infarction | Absent | 1309 | 150 | 0.91 (0.61–1.35) | 0.904 | 0.943 | 1.000 |
| Linezolid + fluoroquinolone | Cerebral infarction | Present | 265 | 37 | 0.74 (0.28–1.98) | 0.904 | 0.943 | 1.000 |
| Linezolid + fluoroquinolone | Hydrocephalus | Absent | 1455 | 156 | 0.88 (0.60–1.28) | 0.918 | 0.943 | 1.000 |
| Linezolid + fluoroquinolone | Hydrocephalus | Present | 119 | 31 | 0.94 (0.30–2.93) | 0.918 | 0.943 | 1.000 |
| Linezolid + fluoroquinolone | MRI complication phenotype | No | 1493 | 172 | 0.89 (0.62–1.27) | 0.607 | 0.943 | 1.000 |
| Linezolid + fluoroquinolone | MRI complication phenotype | Yes | 81 | 15 | 0.04 (<0.01–1.58) | 0.607 | 0.943 | 1.000 |
| Linezolid + fluoroquinolone | Age ≥60 years | <60 years | 1181 | 134 | 0.76 (0.50–1.16) | 0.237 | 0.824 | 1.000 |
| Linezolid + fluoroquinolone | Age ≥60 years | ≥60 years | 393 | 53 | 1.22 (0.61–2.41) | 0.237 | 0.824 | 1.000 |

**Supplementary Table S1b. DILI documented during the index hospitalization**

| **Exposure** | **Subgroup** | **Level** | **n** | **Events** | **OR (95% CI)** | **P interaction** | **FDR P** | **Bonferroni P** |
| --- | --- | --- | --- | --- | --- | --- | --- | --- |
| Linezolid | Drug-resistant tuberculosis | Absent | 1476 | 108 | 1.01 (0.66–1.54) | 0.031 | 0.555 | 0.559 |
| Linezolid | Drug-resistant tuberculosis | Present | 98 | 8 | 0.06 (<0.01–2.10) | 0.031 | 0.555 | 0.559 |
| Linezolid | Drug-resistance gene positivity | Absent | 1519 | 109 | 0.98 (0.64–1.49) | 0.062 | 0.555 | 1.000 |
| Linezolid | Drug-resistance gene positivity | Present | 55 | 7 | NE | 0.062 | 0.555 | 1.000 |
| Linezolid | Cerebral infarction | Absent | 1309 | 100 | 0.88 (0.57–1.37) | 0.914 | 0.968 | 1.000 |
| Linezolid | Cerebral infarction | Present | 265 | 16 | 1.58 (0.40–6.29) | 0.914 | 0.968 | 1.000 |
| Linezolid | Hydrocephalus | Absent | 1455 | 101 | 0.79 (0.51–1.20) | 0.108 | 0.645 | 1.000 |
| Linezolid | Hydrocephalus | Present | 119 | 15 | 2.71 (0.36–20.29) | 0.108 | 0.645 | 1.000 |
| Linezolid | MRI complication phenotype | No | 1493 | 105 | 0.82 (0.54–1.25) | 0.292 | 0.679 | 1.000 |
| Linezolid | MRI complication phenotype | Yes | 81 | 11 | 1.34 (0.18–9.99) | 0.292 | 0.679 | 1.000 |
| Linezolid | Age ≥60 years | <60 years | 1181 | 99 | 0.84 (0.53–1.31) | 0.865 | 0.968 | 1.000 |
| Linezolid | Age ≥60 years | ≥60 years | 393 | 17 | 1.33 (0.36–4.91) | 0.865 | 0.968 | 1.000 |
| Fluoroquinolone | Drug-resistant tuberculosis | Absent | 1476 | 108 | 1.13 (0.75–1.71) | 0.969 | 0.969 | 1.000 |
| Fluoroquinolone | Drug-resistant tuberculosis | Present | 98 | 8 | 0.69 (<0.01–50.32) | 0.969 | 0.969 | 1.000 |
| Fluoroquinolone | Drug-resistance gene positivity | Absent | 1519 | 109 | 1.14 (0.76–1.73) | 0.865 | 0.968 | 1.000 |
| Fluoroquinolone | Drug-resistance gene positivity | Present | 55 | 7 | NE | 0.865 | 0.968 | 1.000 |
| Fluoroquinolone | Cerebral infarction | Absent | 1309 | 100 | 1.22 (0.80–1.86) | 0.332 | 0.679 | 1.000 |
| Fluoroquinolone | Cerebral infarction | Present | 265 | 16 | 0.32 (0.06–1.77) | 0.332 | 0.679 | 1.000 |
| Fluoroquinolone | Hydrocephalus | Absent | 1455 | 101 | 1.16 (0.76–1.76) | 0.725 | 0.968 | 1.000 |
| Fluoroquinolone | Hydrocephalus | Present | 119 | 15 | 0.99 (0.20–4.86) | 0.725 | 0.968 | 1.000 |
| Fluoroquinolone | MRI complication phenotype | No | 1493 | 105 | 1.17 (0.77–1.76) | 0.592 | 0.901 | 1.000 |
| Fluoroquinolone | MRI complication phenotype | Yes | 81 | 11 | 0.37 (0.07–1.93) | 0.592 | 0.901 | 1.000 |
| Fluoroquinolone | Age ≥60 years | <60 years | 1181 | 99 | 1.00 (0.65–1.54) | 0.267 | 0.679 | 1.000 |
| Fluoroquinolone | Age ≥60 years | ≥60 years | 393 | 17 | 1.61 (0.47–5.58) | 0.267 | 0.679 | 1.000 |
| Linezolid + fluoroquinolone | Drug-resistant tuberculosis | Absent | 1476 | 108 | 1.03 (0.67–1.58) | 0.232 | 0.679 | 1.000 |
| Linezolid + fluoroquinolone | Drug-resistant tuberculosis | Present | 98 | 8 | <0.01 (<0.01–1990.05) | 0.232 | 0.679 | 1.000 |
| Linezolid + fluoroquinolone | Drug-resistance gene positivity | Absent | 1519 | 109 | 1.01 (0.66–1.54) | 0.340 | 0.679 | 1.000 |
| Linezolid + fluoroquinolone | Drug-resistance gene positivity | Present | 55 | 7 | NE | 0.340 | 0.679 | 1.000 |
| Linezolid + fluoroquinolone | Cerebral infarction | Absent | 1309 | 100 | 1.09 (0.70–1.70) | 0.167 | 0.679 | 1.000 |
| Linezolid + fluoroquinolone | Cerebral infarction | Present | 265 | 16 | 0.23 (0.02–2.12) | 0.167 | 0.679 | 1.000 |
| Linezolid + fluoroquinolone | Hydrocephalus | Absent | 1455 | 101 | 0.92 (0.59–1.44) | 0.601 | 0.901 | 1.000 |
| Linezolid + fluoroquinolone | Hydrocephalus | Present | 119 | 15 | 0.94 (0.16–5.62) | 0.601 | 0.901 | 1.000 |
| Linezolid + fluoroquinolone | MRI complication phenotype | No | 1493 | 105 | 0.97 (0.63–1.49) | 0.784 | 0.968 | 1.000 |
| Linezolid + fluoroquinolone | MRI complication phenotype | Yes | 81 | 11 | 0.05 (<0.01–0.57) | 0.784 | 0.968 | 1.000 |
| Linezolid + fluoroquinolone | Age ≥60 years | <60 years | 1181 | 99 | 0.86 (0.54–1.36) | 0.462 | 0.832 | 1.000 |
| Linezolid + fluoroquinolone | Age ≥60 years | ≥60 years | 393 | 17 | 1.43 (0.48–4.31) | 0.462 | 0.832 | 1.000 |

Note: For the combined-exposure estimate in the drug-resistant-tuberculosis-positive stratum, the estimate was highly unstable because of sparse events and should not be interpreted clinically. Values below 0.01 are shown as <0.01; NE indicates that the stratum-specific weighted model did not converge.

Abbreviations: CI, confidence interval; DILI, drug-induced liver injury; FDR, false discovery rate; MRI, magnetic resonance imaging; NE, not estimable; OR, odds ratio.
